# Adherence to Infant Feeding Guidelines in the First Foods New Zealand Study

**DOI:** 10.3390/nu15214650

**Published:** 2023-11-02

**Authors:** Kimberley J. Brown, Kathryn L. Beck, Pamela von Hurst, Anne-Louise Heath, Rachael Taylor, Jillian Haszard, Lisa Daniels, Lisa Te Morenga, Jenny McArthur, Rebecca Paul, Emily Jones, Ioanna Katiforis, Madeleine Rowan, Maria Casale, Neve McLean, Alice Cox, Elizabeth Fleming, Bailey Bruckner, Rosario Jupiterwala, Andrea Wei, Cathryn Conlon

**Affiliations:** 1 School of Sport, Exercise and Nutrition, Massey University, Auckland 0632, New Zealand; k.brown1@massey.ac.nz (K.J.B.); k.l.beck@massey.ac.nz (K.L.B.); p.r.vonhurst@massey.ac.nz (P.v.H.); r.paul@massey.ac.nz (R.P.); e.jones@massey.ac.nz (E.J.); m.casale@massey.ac.nz (M.C.); r.p.monzales@massey.ac.nz (R.J.); a.wei@massey.ac.nz (A.W.); 2 Department of Human Nutrition, University of Otago, Dunedin 9016, New Zealand; anne-louise.heath@otago.ac.nz (A.-L.H.); jenrose19@hotmail.com (J.M.); ioanna.katiforis@postgrad.otago.ac.nz (I.K.); maddie.rowan@otago.ac.nz (M.R.); mclne448@student.otago.ac.nz (N.M.); liz.fleming@otago.ac.nz (E.F.); bruba664@student.otago.ac.nz (B.B.); 3 Department of Medicine, University of Otago, Dunedin 9016, New Zealand; rachael.taylor@otago.ac.nz (R.T.); lisa.daniels@otago.ac.nz (L.D.); coxal603@student.otago.ac.nz (A.C.); 4 Biostatistics Centre, University of Otago, Dunedin 9016, New Zealand; jill.haszard@otago.ac.nz; 5 Research Centre for Hauora and Health, Massey University, Wellington 6140, New Zealand; l.temorenga@massey.ac.nz

**Keywords:** infant, feeding guidelines, adherence, breastfeeding, complementary feeding, food group, salt, sugar, beverages

## Abstract

Infant feeding guidelines provide evidence-based recommendations to support optimal infant health, growth, and development, and exploring adherence to guidelines is a useful way of assessing diet quality. The aim of this study was to determine adherence to the recently updated Ministry of Health “Healthy Eating Guidelines for New Zealand Babies and Toddlers (0–2 years old)”. Data were obtained from First Foods New Zealand, a multicentre observational study of 625 infants aged 7.0–10.0 months. Caregivers completed two 24-h diet recalls and a demographic and feeding questionnaire. Nearly all caregivers (97.9%) initiated breastfeeding, 37.8% exclusively breastfed to around six months of age, and 66.2% were currently breastfeeding (mean age 8.4 months). Most caregivers met recommendations for solid food introduction, including appropriate age (75.4%), iron-rich foods (88.3%), puréed textures (80.3%), and spoon-feeding (74.1%). Infants consumed vegetables (63.2%) and fruit (53.9%) more frequently than grain foods (49.5%), milk and milk products (38.6%), and meat and protein-rich foods (31.8%). Most caregivers avoided inappropriate beverages (93.9%) and adding salt (76.5%) and sugar (90.6%). Our findings indicated that while most infants met the recommendations for the introduction of appropriate solid foods, the prevalence of exclusive breastfeeding could be improved, indicating that New Zealand families may need more support.

## 1. Introduction

Rapid dietary changes are observed as an infant transitions from an exclusive milk diet to one that resembles that of the family [1,2,3,4]. To support those involved in the care of infants, guidelines have been developed to provide evidence-based nutrition recommendations. Guidelines provide population health advice on meeting nutrition requirements for growth, development, and the establishment of healthy behaviours [5]. Most infant feeding guidelines include recommendations for breastfeeding, complementary feeding, dietary variety (food groups), and appropriate foods and beverages.

In New Zealand (NZ), the Ministry of Health (MoH) infant feeding guidelines “Healthy Eating Guidelines for New Zealand Babies and Toddlers (0 to 2 years old)” were recently revised and updated to include six statements on breastfeeding and formula feeding, complementary feeding, food groups, appropriate foods and beverages, and feeding environments [5]. These guidelines are available to NZ whānau online and are promoted by Whānau Āwhina Plunket, which provides Well Child services to approximately 85% of NZ infants and their whānau [6]. Mothers are recommended to exclusively breastfeed (EBF) their infant until they are around six months of age, in accordance with the World Health Organization’s (WHO) recommendations [7]. Exclusive breastfeeding has benefits for both mothers and their infants, including the provision of all of the energy and nutrients that an infant needs; it also supports infant gastrointestinal development, reduces the risk of infant mortality, supports mother–infant bonding, and reduces the risk of maternal breast and ovarian cancer [7]. At six months of age, breast milk is not able to provide adequate energy and nutrients, and complementary feeding should be initiated [5]. Iron-rich foods (including meat, poultry, fish, seafood, and iron-fortified infant cereals), vegetables, and fruits are suitable first foods and should each be offered daily once complementary feeding has started. Infants should be offered a wide variety of foods, especially during the first weeks and months, as this helps to establish healthy taste preferences. There is no need to introduce new foods one at a time unless the food is a common allergen. A variety of foods from the main food groups—vegetables and fruit; grain foods; milk and milk products; and legumes, nut butters, eggs, fish, seafood and chicken or lean red meat (referred to as meat and protein-rich foods from here)—should be included in an infant’s diet every day, increasing the amount consumed and food texture as developmentally appropriate [5,8]. Inappropriate foods and beverages, such as foods with added sugar and salt (e.g., confectionery, biscuits, ice cream, processed meats, and fast foods) and beverages other than breast milk, infant formula, and water (such as juice, cordial, fruit drink, flavoured milk, soft drinks, tea, coffee, and alcohol) should be avoided [5].

Investigating adherence to feeding guidelines is a common method of measuring diet quality. Golley et al. [9] were the first to develop and utilise an index that measured adherence to infant feeding guidelines in a high-income country (England). Growing Up in New Zealand (GUiNZ), a longitudinal study of 6470 ethnically diverse infants, measured adherence to the 2008 MoH infant feeding guidelines [10]. The GUiNZ study collected data on infant feeding at nine months of age between 2009 and 2011 and found low levels of adherence (defined as less than 50%) to breastfeeding recommendations [10,11]. Exclusive breastfeeding to around six months of age was met by 35%, while 37% continued to breastfeed to 12 months or beyond. Similar relatively low adherence to EBF recommendations has been observed in other high-income countries [12,13,14,15,16], indicating a global issue. In some studies, low adherence to EBF recommendations can be explained by an early introduction of solid foods [9,10,17], with recommendations for EBF and introducing solid foods often overlapping. In NZ and Australia, recent studies have suggested that the age of introduction of solid foods has increased to align with recommendations [13,18]. The impact this has on EBF adherence is yet to be explored.

Studies have consistently shown high adherence levels for exposure to iron-rich foods, even though assessment methods have varied. In GUiNZ, high levels (80% of infants aged nine months of age) of adherence were found for offering iron-rich foods daily [10], while other studies have reported adherence levels for starting iron-rich foods of 89% and 87% at six [9] and seven [17] months, respectively. By contrast, adherence to beverage recommendations appears to be more variable, depending on what beverages are reported [9,10,17]. In GUiNZ, a moderate (defined as 50–80%) adherence to avoiding inappropriate drinks (61%) was reported at nine months of age [10], with juice reported as the most commonly consumed inappropriate beverage [11,19,20].

Adherence to food group recommendations is typically assessed by the consumption of vegetables, fruit, grain foods, milk and milk products, and meat and protein-rich foods, with varying levels of adherence being found between food groups [9,10,17]. The GUiNZ study found high levels of adherence for the daily consumption of vegetables (91%), fruit (87%), and grain foods (90%) in infants [10,11]. The study also found high adherence levels for the consumption of breast milk or infant formula (96%), although it did not report all milk and milk products [10,11]. Meat and protein-rich foods were less frequently consumed daily (61%). In other high-income countries, similar trends have been observed [9,21]. Although different dietary assessment methods have been used, meat and protein-rich foods are often reported as the least-frequently-consumed food group by infants [9,21]. However, the intake of vegetables and fruits appears to be lower than that reported in NZ [9,17,21]. Mixed results have been found for the offering of foods with added salt and sugar to infants, with high adherence levels for avoiding added sugar (86%) and salt (84%) in GUiNZ [10] but low adherence in the United States of America (USA) [17].

Various sociodemographic characteristics have been associated with adherence to infant feeding guidelines. In GUiNZ, mothers of European ethnicity, of older age, with a higher education, who attended antenatal classes, and who had a partner, as well as those living in the least deprived neighbourhoods, were more likely to meet recommendations [10]. Similar trends have been observed in the USA [22], the United Kingdom (UK) [23,24], Ireland [25], and other European countries [26]. Maternal adherence to adult feeding guidelines [3,21,24] and having fewer children [24,27,28] have also been associated with greater adherence to infant feeding guidelines.

In recent years, there have been notable changes in how infants are fed. Examples of these changes include the increasing availability of commercial infant foods [29,30] and trends surrounding baby-led weaning (BLW) [31,32], where an infant is given whole pieces of food from the start of complementary feeding [18,33]. The MoH does not currently recommend BLW in New Zealand, as more research is required to determine potential risks [5]. The practice also conflicts with recommendations for spoon-feeding puréed foods when starting complementary feeding. The advent of these approaches to infant feeding may have influenced adherence to other guidelines as well.

This study aimed to investigate adherence to the key indicators from the 2021 “Healthy Eating Guidelines for New Zealand Babies and Toddlers (0 to 2 years old)” in infants. Associations between sociodemographic characteristics and adherence to the guidelines were also explored.

## 2. Materials and Methods

### 2.1. Study Design

Data for the current study were obtained as part of the First Foods New Zealand (FFNZ) observational study. A detailed description of FFNZ’s methods can be found elsewhere [34]. FFNZ recruited 625 infants from the Auckland and Dunedin regions of NZ using word of mouth and Facebook advertisements. To ensure sufficient representation of Māori and Pacific infants, advertisements were promoted in Māori and Pasifika people’s community groups. Data were collected between July 2020 and February 2022. Ethical approval was obtained from the Health and Disability Ethics Committee New Zealand (19/STH/151), and written consent was obtained from parent participants before data collection. The study was registered with the Australian New Zealand Clinical Trials Registry (www.anzctr.org.au, accessed on 29 October 2023, registration number: ACTRN12620000459921).

### 2.2. Participants

To meet eligibility criteria, infants were required to live in the Auckland or Dunedin regions of NZ, be 7.0 to 10.0 months of age at the time of participation, and not have taken part in a nutrition intervention study that might have influenced their diet. Caregivers were required to be 16 years or older and able to communicate in English. The screening questionnaire was available on the FFNZ website and generated 1424 responses. Of those, 1198 were eligible, and 630 provided written consent. Five participants did not meet the eligibility criteria, leaving a final sample of 625 infants. Questionnaire data were available from all infants, and two days of diet recall data were available from 614 infants, with the remaining 11 infants providing one day of dietary data.

### 2.3. Data Collection

Participants were invited to attend two study appointments at their home or closest research centre approximately one week apart. Due to COVID-19 restrictions, some second appointments were conducted online. At the first appointment, participants completed a questionnaire investigating demographics, infant health, breastfeeding, and complementary feeding practices. Participants who reported two or more ethnic groups were assigned to a single group using the MoH prioritisation system (order of ranking: Māori, Pacific, Asian, Others, European) [35]. Socioeconomic deprivation levels were assigned according to the NZ Index of Deprivation, which uses the participant’s home address [36] and is displayed as deciles, with low deprivation being classified as deciles 1–3 and high deprivation as deciles 8–10. At each of the two appointments, caregivers completed a 24-h diet recall using a multiple-pass method. Caregivers recalled all breastfeeds, foods, and beverages offered, and the amount consumed the day before their appointment. To aid in portion size estimation, caregivers were asked to take photographs of foods and drinks when they were offered. Measurement prompts (grid sheets (square and circle), measuring cups and spoons, exemplar infant food pouches and jars, infant bowls, and thickness sticks) were also available to guide portion size estimation. Open questions were administered to determine the addition of salt and sugar to infant foods. If infants attended an early child education centre (ECE) or were cared for by someone other than their main caregiver, a food diary with written prompts was provided. Telephone or email contact was made, with caregiver consent, if additional details were required. After their second appointment, caregivers were emailed a final questionnaire, which included the Paediatric Eating Assessment Tool (PediEAT) [37]. Responses for “eats a variety of foods (fruits, vegetables, proteins, etc.) (always, almost always, often, sometimes, almost never, and never)” were used to describe caregiver perceptions of diet variety.

### 2.4. Adherence to National Infant Feeding Guidelines

Indicators presented in this paper were based on the “Healthy Eating Guidelines for New Zealand Babies and Toddlers (0 to 2 years old)” [5]. Recommendations that were measurable from FFNZ data and applied to those aged 7.0 to 10.0 months were selected (Table 1). Mothers reported breastfeeding initiation rates and when breastfeeding was stopped or if they continued to breastfeed. Exclusive breastfeeding to “around six months” [5] was defined as “5 months” or “6 months” (from a pull-down menu of months) being the age when something other than breast milk, i.e., either another drink, or solid foods, was first introduced. Due to the age of infants recruited, breastfeeding to two years of age was not assessed. Instead, current breastfeeding status at the time of participation (7.0 to 10.0 months of age) was reported. Food variety, or the consumption of diverse food groups, was determined by whether each food group was included at least once per day in both diet recalls, in accordance with the MoH recommendation for daily consumption of each food group [5].

### 2.5. Food Coding

All foods consumed in diet recalls (n = 20,975) were individually coded into food groups, using the MoH infant and adult guidelines as a guide (Table 2). The food groups were as follows: vegetables, fruit, grain foods, milk and milk products, and meat and protein-rich foods. Ingredients in recipes or mixed dishes (e.g., baby food pouches) containing multiple food groups were individually assigned to a food group, regardless of the amount consumed. Foods were only assigned to a food group if they complied with the guidelines. This meant that foods such as sour cream and fruit juice, for example, were not considered to contribute to intake from the “milk and milk products” and “fruit” food groups, respectively. Iron-rich foods included meat, poultry, fish, seafood, and iron-fortified infant cereals, as per MoH guidelines [5].

### 2.6. Statistical Analysis

Variables were reported as percentages and means (standard deviation (SD)) from the study population. The proportions of the sample that met the indicator were also adjusted by weighting for ethnicity and socioeconomic deprivation to represent the New Zealand population more closely [38] and estimate adherence with a 95% logit-transformed confidence interval. Logistic regression was used to estimate odds ratios, 95% confidence intervals, and *p*-values for associations between sociodemographic characteristics and adherence to different indicators, and *p*-values < 0.05 indicated statistical significance. Odds ratios were only calculated for variables with at least 10 participants in each cell and indicators with more than 10% of the sample in each category. Associations between sociodemographic variables (caregiver age, education, employment status; maternal parity; number of children living in household; use of childcare/ECE; and socioeconomic deprivation) and MoH recommendations (exclusive breastfeeding, current breastfeeding, solids introduction between 5 and 7 months, puréed foods introduced first, iron-rich foods introduced first, food variety (iron-rich foods, vegetables, and fruit consumed on both recall days), no salt or sugar added, and infants not fully spoon-fed at current age) were investigated. Analyses were performed using Stata Statistical Software (version 17, StataCorp LP, College Station, TX, USA) and Microsoft Excel (version 16.66).

## 3. Results

### 3.1. Maternal and Infant Characteristics

Maternal and infant demographic characteristics are summarised in Table 3. Infants and caregivers were on average (mean (SD)) 8.4 (0.8) months and 32.7 (4.9) years of age, respectively. The majority (98%) of caregivers were the infant’s mother. Six respondents were fathers, one was a grandparent, and one was a guardian.

### 3.2. Adherence to Infant Feeding Guidelines

Adherence to the MoH infant feeding guidelines (Table 1) is shown in Table 4, with further detail regarding each indicator and sociodemographic predictors provided below. The proportions of the sample that met the indicator were also weighted for ethnicity and socioeconomic deprivation in Table 4 to more closely represent the New Zealand population [38], allowing wider conclusions to be made.

#### 3.2.1. Breastfeeding

**Figure 1:**
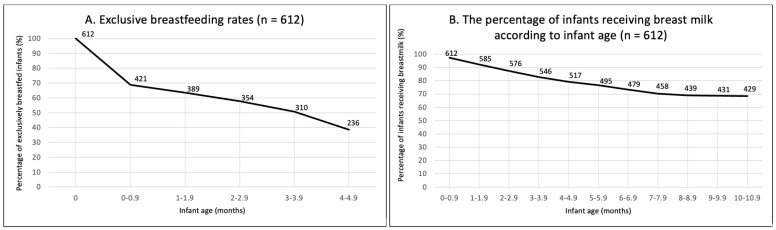
(**A**) Exclusive breastfeeding prevalence (between birth and 4.9 months). (**B**) The percentage of infants receiving breast milk according to infant age. N = 612.

Breastfeeding had been initiated by 97.9% of women. At around six months of age, 37.8% met EBF recommendations (Table 4). Of those who did not meet the recommendations, 51% stopped breastfeeding before one month of age (Figure 1A). At the time of participation, 66.2% were still breastfeeding. A progressive decline in breastfeeding was observed between birth and seven months; however, it seemed to be stabilising around 9–10 months of age (Figure 1B).

Infants had a higher odds of meeting EBF recommendations if their caregivers had the following characteristics: they were older (1.05 (1.01, 1.08), *p* = 0.008) vs. young; their highest qualification was university vs. school (0.37 (0.22, 0.64), *p* < 0.001); they were not currently working vs. employed full-time (0.54 (0.31, 0.96), *p* = 0.035); they were multiparous mothers vs. primiparous (0.67 (0.48, 0.93), *p* = 0.016); they had two children in the household (1.56 (1.08, 2.27), *p* = 0.019) vs. one child; and they did not use ECE vs. those who did (0.60 (0.38, 0.95), *p* = 0.028) (Table 5).

Caregivers were more likely to be breastfeeding at the time of the study (mean infant age 8.4 months) if their highest qualification was university vs. school (0.41 (0.25, 0.64), *p* < 0.001) or polytechnic (0.47 (0.31, 0.72), *p* < 0.001) only; they were not currently working vs. employed full-time (0.41 (0.25, 0.64), *p* = 0.014); they had two children (1.52 (1.03, 2.26), *p* = 0.037) vs. one child; their infant did not attend ECE vs. those whose infant did attend ECE (0.54 (0.35, 0.81), *p* = 0.004); or they lived in low vs. high socioeconomic deprivation areas (0.61 (0.39, 0.96), *p* = 0.032).

#### 3.2.2. Introduction of Solids

**Figure 2:**
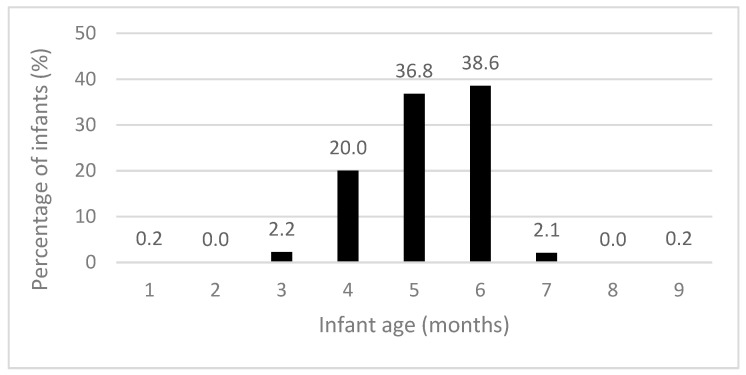
Infant age when solid foods were introduced (n = 625).

The recommendation for introducing solid foods “around” six months of age was met by 75.4% of participants (Table 4). Solid foods were introduced at a mean age (SD) of 5.18 (0.89) months, with the majority starting by six months (Figure 2). Most infants met the recommendations for puréed food textures (80.3%), spoon-feeding (74.1%), and the introduction of iron-rich foods (88.3%) (Table 4). Few infants consumed an iron-rich food, vegetables, and fruit during both diet recall days (13.4%). During the time when solid foods were introduced, 61.4% and 65.6% of infants consumed infant rice cereal and red meat, respectively.

Infants were more likely to be introduced to solid foods “around” six months of age if they had younger (1.12 (1.08, 0.02), *p* < 0.001) vs. older caregivers; their caregiver’s highest qualification was university level vs. school (0.21 (0.13, 0.34), *p* < 0.001) or polytechnic (0.53 (0.33, 0.84), *p* = 0.007); their caregiver had one child in the household vs. four or more (0.44 (0.23, 0.84), *p* = 0.013); they did not attend ECE vs. those who did (0.54 (0.35, 0.81), *p* = 0.004); or they were living in a low vs. high socioeconomic deprivation area (0.47 (0.28, 0.77), *p* = 0.003) (Table 6). Infants were more likely to be spoon-fed when starting solid foods if their caregiver’s highest qualification was school-level (2.08 (1.16, 3.70), *p* = 0.013) or polytechnic-level (1.70 (1.05, 2.77), *p* = 0.032) vs. university-level and if the infant attended ECE (2.29 (1.30, 4.03), *p* = 0.004) vs. those who did not.

#### 3.2.3. Food Group Intake of Infants

Vegetables (63.1%) and fruit (53.9%) were the most commonly consumed food groups. Fewer than half of infants consumed grain foods (49.5%), milk and milk products (38.6%), and meat and protein-rich foods (31.8%) on both diet recall days. A small proportion (6.5%) of infants consumed each food group on both diet recall days. When asked if caregivers felt that their infant consumed a variety of foods, 144 of 146 (98.6%) participants reported “sometimes or more”.

Infants were more likely to consume iron-rich foods and vegetables on both diet recall days if caregivers were older (iron-rich: 1.06 (1.02, 1.10), *p* = 0.003; vegetables: 1.07 (1.04, 1.12), *p* <0.001) vs. younger, primiparous (iron-rich: 1.44 (1.01, 2.04), *p* = 0.042; vegetables: 1.81 (1.29, 2.52), *p* = 0.001) vs. multiparous, and had a university qualification as their highest level of education vs. a polytechnic qualification (iron-rich: 0.58 (0.36, 0.94), *p* = 0.020; vegetables: 0.52 (0.35, 0.79), *p* < 0.001). Infants were also more likely to consume vegetables on both diet recall days if the caregiver’s highest qualification was university vs. school (0.42 (0.27, 0.68), *p* < 0.001), there was one child in the household vs. three (0.53 (0.33, 0.86), *p* = 0.010) and four or more (0.23 (0.12, 0.45), *p* < 0.001), and they lived in an area of low vs. high socioeconomic deprivation (0.45 (0.29, 0.70), *p* < 0.001) (Table 7). Infants were more likely to consume fruit if their caregiver’s highest qualification was university vs. school (0.47 (0.29, 0.75), *p* = 0.002).

#### 3.2.4. Appropriate Foods and Beverages

Most participants met the recommendations for avoiding inappropriate foods, including salt (75.7%) and sugar (90.9%) (Table 4). For those using salt, this was typically added to roasted meat/vegetables, vegetable mash, family meals, and potato fries. Sugar was added to Weet-Bix, baking, fruit purées, and family meals. Inappropriate beverages were rarely consumed, and 93.9% of infants met the recommendation for not offering juice, cordial, fruit drink, flavoured milk, soft drinks, tea, coffee, or alcohol (Table 4). Juice was the predominant inappropriate beverage reported in diet recalls (6.6%). Tea and coffee were not reported in any diet recall.

The associations between sociodemographic characteristics and not adding salt and sugar to infant food are shown in Table 8. Primiparous mothers (0.53 (0.36, 0.78), *p* = 0.001) were less likely to add salt to infant foods than multiparous mothers. Those with two (1.64 (1.06, 2.54), *p* = 0.026) or three (1.87 (1.09, 3.18), *p* = 0.022) children in the household were more likely to add salt to infant foods than those with one child. Younger caregivers (0.94 (0.88, 0.99), *p* = 0.019) were less likely to add sugar than older caregivers, and those with a school qualification only (3.20 (1.65, 6.19), *p* = 0.001) were more likely to add sugar than university-educated caregivers.

#### 3.2.5. Feeding Environment

At the age of participation, 86% of infants were no longer fully spoon-fed, aligning with the MoH recommendation that encourages self-feeding from a young age (Table 4). Infants were more likely to meet the recommendation for self-feeding if there was one child in the household vs. four or more children in the household (0.41 (0.20, 0.87), *p* = 0.020) (Table 9). Infants of caregivers with part-time employment had a higher odds of self-feeding than those whose caregivers were not currently working (2.21 (1.10, 4.44), *p* = 0.026).

## 4. Discussion

The current study has provided insight into adherence to the 2021 NZ MoH infant feeding guidelines in an ethnically diverse infant population. High levels of adherence to recommendations for the introduction of solid foods, appropriate foods and beverages, and eating environments were observed, whereas breastfeeding and food group recommendations were less commonly met. Socioeconomic deprivation, caregiver education, parity, and the number of children in the household were key sociodemographic characteristics associated with adherence to the NZ guidelines.

### 4.1. Adherence to Breastfeeding Recommendations

Breastfeeding initiation rates in NZ are high [3,19]; however, as seen in this study and previous NZ research, many women do not meet EBF and continued breastfeeding recommendations [10,15,16,39,40]. Although most women did not meet the EBF recommendation, our cohort’s response was higher (37.8%) than that observed in GUiNZ (34.3%) in 2009–2011, suggesting that adherence might be improving. Despite higher adherence, further promotion is required if NZ is to meet the 2030 global nutrition target of at least 70% of women EBF to six months of age [41]. A possible area to target is EBF during the first month—the vast majority of those who did not meet the EBF recommendation in our study stopped during this time. These results indicate a crucial time window that needs further investigation. Although we did not investigate the reasons why women stopped EBF in our study, we found that primiparous women were less likely to meet the EBF recommendation. Similar patterns have been observed in NZ [10] and other high-income countries [42], with strong associations between EBF and previous breastfeeding experience [42]. Early intervention through breastfeeding support programmes has shown beneficial outcomes [43] and could be targeted towards those, such as primiparous mothers, who are at higher risk of not meeting EBF recommendations. We also observed that caregivers who were older, more highly educated, not currently working, and whose infants did not attend ECE were more likely to meet the EBF recommendation. This agrees with global findings [44], with factors such as education, employment status, and use of ECE often being interconnected with socioeconomic status, with those who are more highly educated having greater opportunity to take paid maternity leave than those who are less highly educated and require ECE support when returning to work [42,45]. Sustaining breastfeeding is typically harder for women who return to work in the first twelve months postpartum, with common reports of inadequate support and facilities for breastfeeding [46,47]. Finding ways to support women who are required to return to work, such as encouraging peer support and introducing regulations for the provision of appropriate facilities, has improved the incidence of EBF in other countries [48] and would be beneficial in NZ. Further—qualitative—input from mothers would also be helpful to determine what support is required to achieve the EBF recommendations.

After six months of age, breastfeeding continued to decline. Rates of decline, however, seem to have reduced since those reported in GUiNZ (48%) [11] and to have plateaued around 9–10 months of age, with 70.4% of women (n = 431) continuing to breastfeed at nine months of age in our study. As in GUiNZ [10], women in the current study with higher levels of education were more likely to meet the continued breastfeeding recommendations. As shown for EBF, education, returning to work, use of ECE, and socioeconomic status are often interconnected, making these complex markers to investigate [42,45]. The positive influence of previous experience was also evident for continued breastfeeding, as seen for EBF, with caregivers of two children having higher adherence, suggesting that experience and education are key factors in sustaining breastfeeding. Further support to sustain breastfeeding for first-time mothers and those who are required to return to work may improve rates of adherence in NZ.

### 4.2. Adherence to Complementary Feeding Recommendation

The recommendation to introduce solid foods “around six months of age” was met by most FFNZ participants (75.4%, average age of 5.18 months); this was notably higher than for participants in the GUiNZ study (56.9%), which reported that 39.4% of infants had started solid foods before or at four months of age [10,49]. Other recent NZ studies have also suggested that the average age of solid food introduction has increased since GUiNZ, with an average age of 5.2 months reported in 2018 [18] and few infants being offered solid foods before four months (5.4%) in the 2020–2021 NZ Health Survey [50]. However, sample bias within studies focusing on nutrition may explain differences, with a common limitation being that those who are interested in nutrition take part in those studies, compared to GUiNZ, which included a wider range of health and environmental variables beyond nutrition. A higher number of Pacific mothers, who were less likely to meet solid food introduction recommendations than European mothers, also took part in GUiNZ compared to the current study. Similar trends exist in Australia, with survey results suggesting that the average age of solid food introduction is moving towards “around six months” (5.3 months [3], 5.1 months [51], 5.0 months [13]), with few caregivers providing solid foods before four months [51]. As in GUiNZ, caregivers with lower education levels and those living in areas of high socioeconomic deprivation were less likely to meet this recommendation [10], identifying key population groups who require additional support. The relationship between low adherence and attending ECE was a new finding and may suggest an area of influence that needs additional investigation.

Previously reported trends for the increasing number of caregivers following BLW [52] did not appear to influence food textures or feeding methods in the current study. Most infants consumed puréed foods when solids were introduced and were spoon-fed by an adult. However, university-educated women were less likely to spoon feed their infant when starting solid foods, agreeing with findings that maternal education may influence BLW trends [53]. Attendance at ECE appears to be a positive influence for spoon-feeding, a finding which has not been reported before. The rate of introduction of iron-rich foods when starting solids (88.3%) was similar to that observed in recent studies [18], suggesting that many infants are offered iron-rich foods in the early complementary feeding period. We also observed a higher likelihood that iron-fortified infant cereals were consumed (64.1%) than that reported in a recent online survey (12%) [18] and GUiNZ (47.9%) [11]. This may be secondary to the increased availability of these products in NZ [29]. However, the continuation of offering iron-fortified cereals in addition to other food groups was low, with only 13.4% of infants consuming an iron-rich food, vegetables, and fruit on both recall days. Further promotion of the importance of offering these foods daily during complementary feeding is required in NZ to increase diet diversity and the provision of a wide range of nutritious foods.

### 4.3. Adherence to Food Group Recommendations

Few infants consumed a food from each food group on both diet recall days (6.5%), increasing the risk of nutritional inadequacies. Our results were lower than those reported by GUiNZ [11]. In GUiNZ, most infants consumed vegetables (91% vs. 63.2% in the current study), fruit (87% vs. 53.9%), grain foods (90% vs. 49.5%), and meat and protein-rich foods (61% vs. 31.8%) daily at nine months of age [10,11]. GUiNZ did not report the consumption of milk and milk products. The different (and mainly younger) infant ages we recruited could be a factor that explains why fewer infants met the recommendations in our study, with foods often being gradually added to infants’ diets during the complementary feeding transition [54]. The difference of approximately one month between average ages (8.4 months in the current study vs. 9.0 months in GUiNZ) in the studies, therefore, must be considered. This, however, was not the case when comparing our results to those reported by Morison et al. [33], in which the majority of 6–8-month-old infants consumed vegetables (96%) and fruit (96%) at least once during a three-day food diary. Again, however, differences in methodological approaches need to be considered: in the Morison et al. study [33], intake was calculated across a three-day period, which is likely to have contributed to higher results than when assessing daily intake by 24-h recall, as in our study. Therefore, this is the first study to suggest that most infants aged 7.0 to 10.0 months are not meeting MoH food group recommendations, and further investigation into adherence of infants at different ages is required. Investigation into the nutritional implications of not meeting MoH recommendations is also required to determine the impact that current dietary practices have on nutrient intake. Given that 98.6% of parents reported that their infants consumed a varied diet “sometimes or more”, further promotion about the optimal provision of food groups is likely to be required to support understanding of the recommendations promoting daily consumption of each food group from the start of complementary feeding.

### 4.4. Adherence to Appropriate Food and Beverage Recommendations

Research on the use of added salt and sugar in the infant’s diet has provided conflicting results in NZ. The absence of sugar added to food in the current study (90.6%) is the highest adherence observed in NZ to date, higher than in GUiNZ (86%) [10] and in Morison et al. (55%) [33]. Adherence to the salt recommendation, however, is lower (76.5%) than in GUiNZ (84%) [9] but higher than that reported by Morison et al. (22%) [33]. In the current study, the addition of salt appeared to come from family meals, becoming more frequent with higher parity. This is consistent with results observed in other high-income countries [3,4,55,56,57], indicating a need for education about sources of salt to improve recommendation adherence. Sugar was added to a range of foods, indicating that promotion strategies should focus on key messages (e.g., that added sugar is not required in an infant’s diet).

Before the current study, GUiNZ was the only study that had reported inappropriate drinks consumed by NZ infants [10]. The GUiNZ study found a moderate adherence (61%) to recommendations, whereas adherence in the current study was considerably higher (93.9%). In alignment with GUiNZ [10] and studies from the UK [42], juice continues to be the most commonly consumed inappropriate drink. Further research into why these beverages continue to be offered would allow targeted approaches to improve recommendation adherence.

### 4.5. Strengths, Limitations, and Future Research

The present findings should be interpreted within the context of the study’s strengths and limitations. While the FFNZ study did not have a representative sample, the cohort recruited was diverse in many respects, including a large number of usually unrepresented participants, including the Māori population and those living in higher deprivation areas. Weighted estimates have been provided where appropriate. Our results provide important updated information about adherence to the MoH infant feeding guidelines. A potential limitation is that the key indicators used in this study are those from the 2021 guideline release, and our data were collected between June 2020 and February 2022; therefore, a portion of the reported data were collected before the guidelines were available to the public. The decision to compare to the 2021 guidelines was made after comparisons between guidelines showed minimal changes to the key indicators discussed. Although a full analysis of breastfeeding to two years could not be completed, FFNZ provides a snapshot of “continued breastfeeding” from 7.0 to 10.0 months of age. A small number of infants were born preterm (n = 46), and it is not known whether caregivers reported corrected age for starting solid foods or not. A three-pass assessment approach and photographic memory prompts were used to ensure accuracy of diet recalls, but the 24-h diet recalls relied on caregiver-reported data and have the potential for misreporting. Although a full representation of the diet, including the usual intake of foods, cannot be achieved from two diet recalls, low daily consumption of MoH food groups is evident.

Further research is now required to investigate why early breastfeeding cessation is occurring and what interventions can be implemented to support breastfeeding. Investigation into the nutritional implications of not meeting food group recommendations during complementary feeding will be important to determine the seriousness of these dietary shortcomings.

## 5. Conclusions

The current study results provide an important update on adherence to the newest MoH infant feeding guidelines in NZ. The initiation of breastfeeding continues to be high; however, EBF to “around” six months of age is low. Most infants met recommendations for starting solid foods, but many did not consume foods from the MoH food groups daily. The consumption of inappropriate foods and beverages has improved since previous assessments; however, a small number of caregivers continue to offer salt, sugar, and inappropriate beverages. As seen previously, there are associations between sociodemographic characteristics and adherence, identifying key groups (primiparous mothers, caregivers with lower levels of education, those living with multiple children, and those living in areas of high socioeconomic deprivation) that require additional support with infant feeding. Further research is now required to identify reasons for these findings and the most appropriate support for NZ whānau so that they can adhere to current recommendations.

## Figures and Tables

**Table 1:** Indicators developed from the Ministry of Health infant feeding guidelines [5].

Indicator	Guideline Recommendation	FFNZ Question	FFNZ Indicator
Breastfeeding			
Exclusive breastfeeding duration	Aim to exclusively breastfeed your baby until they are around six months of age.	Questionnaire: “How old was your baby when they first had anything to drink that wasn’t breast milk?” Possible answers: They did not have any breast milk, breast milk is the only drink my baby has had so far, less than 1 month old, 1 month old, 2 months old, 3 months old, 4 months old, 5 months old, 6 months old, 7 months old, 8 months old, 9 months old, 10 months old, 11 months old and “How old was your baby when they first had solid foods?” Possible answers: They haven’t had solids yet, less than 1 month old, 1 month old, 2 months old, 3 months old, 4 months old, 5 months old, 6 months old, 7 months old, 8 months old, 9 months old, 10 months old, 11 months old and “How old was your baby when they first had infant formula?” Possible answers: Less than 1 month old, 1 month old, 2 months old, 3 months old, 4 months old, 5 months old, 6 months old, 7 months old, 8 months old, 9 months old, 10 months old, 11 months old	“5 months” or “6 months”
Breastfeeding duration	Continue to breastfeed for up to two years or longer.	Questionnaire: “Is baby still being breastfed?” Possible answers: yes, no	“Yes”
Introduction of solids			
Age of solids introduction	Around six months of age, when your baby is showing signs of readiness, introduce complementary foods.	Questionnaire: “How old was your baby when they first had solid foods?” Possible answers: They haven’t had solids yet, less than 1 month old, 1 month old, 2 months old, 3 months old, 4 months old, 5 months old, 6 months old, 7 months old, 8 months old, 9 months old, 10 months old, 11 months old	“5 months” or “6 months”
Appropriate foods introduced	Offer iron-rich foods, vegetables and fruit as first foods, and continue to offer these foods every day.	Diet recall data	An iron-rich food ^a^, vegetables, and fruit consumed on both recall days
Appropriate food textures	Start with spoon-fed purées, then progress over the next few weeks to mashed/lumpy foods and soft finger foods.	Questionnaire: “What texture was the first food you gave your baby?” Possible answers: puréed, mashed, chopped, finger food, other	“Puréed”
Appropriate feeding style	Start with spoon-fed purées, then progress over the next few weeks to mashed/lumpy foods and soft finger foods.	Questionnaire: “How was your baby fed when they first started eating solids?” Possible answers: spoon-fed by an adult, mostly spoon-fed by adult, some baby feeding themselves, about half spoon-fed by an adult and half baby feeding themselves, mostly baby feeding themselves, some spoon-feeding by an adult, baby feeding themselves	“Spoon-fed by an adult”
Eating a variety of foods			
Food variety	Once you have started complementary feeding, offer your baby or toddler a variety of nutritious foods every day, including: Vegetables and fruit Grain foods (e.g., iron-fortified infant cereal, oats (porridge), bread, rice, noodles, and pasta) Milk and milk products (e.g., yoghurt and cheese) Meat and protein-rich foods (e.g., lentils, tofu, beans, nut butters, eggs, fish, seafood, chicken, or lean red meat).	Diet recall data	Selected food group offered during both diet recall days
Appropriate foods			
Addition of salt	When preparing food for your baby or toddler, do not add salt.	Diet recall question: “Is salt added to any foods or drinks that baby eats (including on days not covered by the 24-h recall)” Possible answers: yes, no. If yes, what foods/drinks?	“No”
Addition of sugar	When preparing food for your baby or toddler, do not add sugar.	Diet recall question: “Is sugar added to any foods or drinks that baby eats (including on days not covered by the 24-h recall)” Possible answers: yes, no. If yes, what foods/drinks?	“No”
Appropriate drinks	Recommended drinks for your baby or toddler are breast milk ^b^ and water (once they are eating complementary foods). Cow’s milk can be offered as a drink from 12 months of age.	Diet recall question: “Is baby offered any drinks other than breast milk, formula, or water (things like cow’s milk, other milk, juice, soft drinks, tea, alcohol or any other drink)?” Possible answers: yes, no. If yes, what drinks?	“No”
Feeding environment			
Self-feeding	From a young age, encourage your child to feed themselves.	Questionnaire: “How is your baby being fed solids now?” Possible answers: spoon-fed by an adult, mostly spoon-fed by adult, some baby feeding themselves, about half spoon-fed by an adult and half baby feeding themselves, mostly baby feeding themselves, some spoon feeding by an adult, baby feeding themselves, baby does not eat solids	“Mostly spoon-fed by adult” or “some baby feeding themselves” or “about half spoon-fed by an adult and half baby feeding themselves” or “mostly baby feeding themselves” or “some spoon feeding by an adult” or “baby feeding themselves”

^a^ including meat, poultry, fish, seafood, and iron-fortified infant cereals, ^b^ or, if necessary, a commercial infant formula until 12 months of age.

**Table 2:** Foods included and excluded in food group analyses.

Food/Group	Foods Included	Foods Not Included
Vegetables	All vegetables (canned, frozen, fresh, commercial infant foods).	Potato chips, potato fries, herbs, spices, cakes/muffins/slices/biscuits/pancakes/scones including vegetables, dried/freeze-dried vegetable snack foods.
Fruit	All fruit (canned, frozen, fresh, commercial infant foods).	Fruit juice, fruit jam, cakes/muffins/slices/biscuits/pancakes/scones including fruit, dried/freeze-dried fruit snack foods.
Grain foods	Pasta/noodles, rice, bread, cereals, infant cereals, crackers/crispbreads/rice and corn cakes (plain), oats, couscous, semolina, polenta, bulgur, quinoa, buckwheat, muesli, popcorn (plain).	Grain/corn chips, popcorn (butter/sweetened), crackers/crispbreads/rice and corn cakes (flavoured/yoghurt coated), all biscuits, slices, scones, waffles, pikelets/pancakes, bars (muesli bars and other), cakes, pastries, muffins, discretionary snack foods and commercial infant extruded snacks.
Milk and milk products	Cow’s milk ^a^, calcium-fortified plant-based milks ^ab^, cow’s milk cheese and yoghurt, homemade custard.	Breast milk, infant formula, sour cream, cream, non-calcium-fortified plant-based milks ^a^, ice cream, butter, sweetened milk puddings.
Meat and protein-rich foods	Beef, veal, lamb, mutton, fish, seafood, venison, egg, offal meats, pork, legumes, beans, lentils, nuts, seeds, tofu, tahini, hummus, Quorn, pea-protein products.	Pies, sausages, ham, bacon.
Commercial infant foods	Pouches, cans, jars, microwaveable bowls, rusks, cereals, extruded snacks, dried/freeze-dried fruit and vegetable snack foods, baby cereal bars, baby biscuits, discretionary snack foods.	

^a^ included in foods, ^b^ calcium-fortified milks were defined as milks with equal to or higher than 100 mg of calcium per 100 mL of milk (included in foods only) [5].

**Table 3:** Demographic characteristics of infants and caregivers (n = 625).

Demographic Variable		Descriptive Statistic
Infant		
Ethnicity, n (%)	Māori	131 (21.0)
	Pacific	44 (7.0)
	European	344 (55.0)
	Asian	90 (14.4)
	Others	16 (2.6)
Sex ^a^	Male	335 (53.6)
Pre-term birth, n (%) ^b^		46 (7.4)
Caregiver		
Highest qualification, n (%) ^c^	School	93 (14.9)
	Polytechnic or similar	126 (20.2)
	University	405 (64.9)
Maternal parity, n (%)	Primiparous	304 (48.7)
Employment status, n (%)	Employed full-time	70 (11.2)
	Employed part-time	137 (21.9)
	Other ^d^	418 (66.9)
Environment		
Number of children living in household, n (%)	One	284 (45.5)
	Two	200 (32.1)
	Three	95 (15.2)
	Four or more	45 (7.2)
Number of adults living in household, n (%)	One	25 (4.0)
	Two	517 (82.7)
	Three	42 (6.7)
	Four or more	41 (6.6)
Childcare (ECE) used outside of home, n (%)		109 (17.4)
Area-level socioeconomic deprivation, n (%) ^e^	1–3 (low)	181 (29.0)
	4–7	282 (45.1)
	8–10 (high)	162 (25.9)

^a^ infant sex was not specified for one participant, ^b^ born before 37 weeks gestation, ^c^ highest qualification was not specified for one caregiver, ^d^ includes paid parental leave, unpaid parental leave, and unemployed, ^e^ defined according to deciles of the NZ Index of Deprivation using participant home address [36].

**Table 4:** Summary of adherence to indicators developed from the Ministry of Health infant feeding guidelines (n = 625).

Recommendation	Indicator ^a^	n (%) Who Met Recommendation	Adjusted Proportion (95% CI) ^b^ Who Met Recommendation
Breastfeeding			
Aim to exclusively breastfeed your baby until they are around six months of age.	Exclusive breastfeeding to at least 5 and fewer than 7 months	236 (37.8)	38.7 (34.7, 42.7)
Continue to breastfeed for up to two years or longer.	Current breastfeeding at time of participation	414 (66.2)	67.6 (63.7, 71.3)
Introduction of solids			
Around six months of age, when your baby is showing signs of readiness, introduce complementary foods.	Solid food introduced between 5 and fewer than 7 months of age	471 (75.4)	77.4 (73.9, 80.6)
	Puréed-texture food was used when solid foods were introduced	502 (80.3)	80.6 (77.2, 83.6)
	Spoon-fed by an adult when solid foods were introduced	463 (74.1)	74.1 (70.4, 77.5)
	Iron-rich foods introduced when solid foods were introduced	552 (88.3)	88.7 (85.9, 91.0)
	Iron-rich foods, vegetables and fruit consumed daily during recall days	82 (13.4)	- ^d^
Food variety			
Once complementary feeding has started, offer your baby or toddler a variety of nutritious foods every day, including: Vegetables and fruit Grain foods Milk and milk products Meat and protein-rich foods.	Vegetables consumed daily during recall days ^c^	388 (63.2)	- ^d^
	Fruit consumed daily during recall days ^c^	331 (53.9)	- ^d^
	Grain foods consumed daily during recall days ^c^	304 (49.5)	- ^d^
	Milk and milk products consumed daily during recall days ^c^	237 (38.6)	- ^d^
	Meat and protein-rich foods consumed daily during recall days ^c^	195 (31. 8)	- ^d^
Appropriate foods			
When preparing food for your baby or toddler, do not add salt or sugar. When preparing food for your baby or toddler, do not add salt or sugar.	Salt was not added to foods since solid foods were introduced ^e^	455 (75.7)	75.1 (71.3, 78.6)
	Sugar was not added to foods since solid foods were introduced ^e^	546 (90.9)	90.8 (88.1, 93.0)
Recommended drinks for your baby or toddler are breast milk ^f^ and water (once they are eating complementary foods). Cow’s milk can be offered as a drink from 12 months of age. Do not give your baby or toddler juice, cordial, fruit drink, flavoured milk, soft drinks, tea, coffee or alcohol.	Only offered breast milk, infant formula, and/or water at time of participation	587 (93.9)	94.5 (92.3, 96.1)
Feeding environment			
From a young age, encourage your child to feed themselves.	Infants were not 100% spoon-fed at time of participation	543 (86.9)	86.1 (82.8, 88.8)

^a^ how guidelines were assessed in FFNZ, ^b^ proportion who met the indicator, weighted for ethnicity and NZ Index of Deprivation using participant home address [36], ^c^ assessed from 614 participants, ^d^ weighted estimates not provided for adherence of diet recalls, ^e^ assessed from 601 participants, ^f^ or, if necessary, a commercial infant formula until 12 months of age.

**Table 5:** Sociodemographic characteristics associated with adherence to breastfeeding recommendations.

	Exclusively Breastfed to Around 6 Months		Current Breastfeeding	
	Met Recommendation n = 236	Odds Ratio (95% CI)	Met Recommendation n = 414	Odds Ratio (95% CI)
Parent/caregiver age, mean (SD) years				
	33.3 (4.5)	**1.05 (1.01, 1.08)**	32.9 (4.6)	1.03 (1.00, 1.07)
Highest parent/caregiver qualification, n (%)				
School	20 (21.3)	**0.37 (0.22, 0.64)**	49 (52.1)	**0.41 (0.25, 0.64)**
Polytechnic or similar	46 (36.8)	0.80 (0.53, 1.22)	70 (56.0)	**0.47 (0.31, 0.72)**
University	170 (42.0)	Reference	295 (72.8)	Reference
Employment status of parent/caregiver, n (%)				
Employed full-time	18 (25.7)	**0.54 (0.31, 0.96)**	49 (52.1)	**0.41 (0.25, 0.64)**
Employed part-time	55 (40.2)	1.05 (0.71, 1.56)	70 (56.0)	0.47 (0.31, 0.72)
Other ^a^	163 (39.0)	Reference	295 (72.8)	Reference
Maternal parity, n (%)				
Primiparous	100 (33.0)	**0.67 (0.48, 0.93)**	223 (69.5)	0.74 (0.53, 1.03)
Multiparous	136 (42.4)	Reference	190 (62.7)	Reference
Number of children living in household, n (%)				
One	96 (33.9)	Reference	181 (64.0)	Reference
Two	89 (44.5)	**1.56 (1.08, 2.27)**	146 (73.0)	**1.52 (1.03, 2.26)**
Three	38 (40.0)	1.30 (0.80, 2.10)	63 (66.3)	1.11 (0.68, 1.81)
Four or more	13 (28.3)	0.77 (0.39, 1.53)	24 (52.2)	0.61 (1.39, 2.26)
Childcare (ECE) outside the home, n (%)				
No	205 (39.7)	Reference	355 (68.8)	Reference
Yes	31 (28.4)	**0.60 (0.38, 0.95)**	59 (54.1)	**0.54 (0.35, 0.81)**
Socioeconomic deprivation, n (%) ^b^				
1–3 (low)	73 (40.6)	Reference	125 (69.4)	Reference
4–7	106 (37.6)	0.88 (0.60, 1.29)	194 (68.8)	0.97 (0.65, 1.45)
8–10 (high)	57 (35.0)	0.79 (0.51, 1.22)	95 (58.3)	**0.61 (0.39, 0.96)**

Did not meet recommendation: exclusive breastfeeding (n = 389), continued breastfeeding (n = 211). Odds ratio (95% CI) values with *p* values < 0.05 are bolded. ^a^ includes paid parental leave, unpaid parental leave, and unemployed, ^b^ defined according to the NZ Index of Deprivation using participant home address [36].

**Table 6:** Sociodemographic characteristics associated with adherence to solid food introduction recommendations.

	Introduction of Solids between 5 and <7 Months		Puréed Food Texture Introduced First		Spoon-Fed by Adult When Solids First Introduced		Iron-Rich Foods Introduced First	
	Met Recommendation n = 471	Odds Ratio (95% CI)	Met Recommendation n = 502	Odds Ratio (95% CI)	Met Recommendation n = 463	Odds Ratio (95% CI)	Met Recommendation n = 552	Odds Ratio (95% CI)
Parent/caregiver age, mean (SD) years								
	33.4 (4.6)	**1.12 (1.08, 1.16)**	32.7 (4.8)	1.0 (0.96, 1.04)	32.6 (5.0)	0.99 (0.95, 1.02)	32.6 (4.9)	0.99 (0.94, 1.04)
Highest parent/caregiver qualification								
School	47 (50.0)	**0.21 (0.13, 0.34)**	75 (79.8)	0.99 (0.56, 1.73)	78 (83.0)	**2.08 (1.16, 3.70)**	84 (89.4)	1.08 (0.52, 2.22)
Polytechnic or similar	89 (71.2)	**0.53 (0.33, 0.84)**	103 (82.4)	1.18 (0.70, 1.98)	100 (80.0)	**1.70 (1.05, 2.77)**	108 (86.4)	0.81 (0.45, 1.48)
University	334 (82.5)	Reference	324 (80.0)	Reference	284 (70.1)	Reference	359 (88.6)	Reference
Employment status of parent/caregiver								
Employed full-time	49 (70.0)	0.68 (0.39, 1.19)	56 (80.0)	1.03 (0.55, 1.95)	56 (80.0)	1.54 (0.82, 2.87)	58 (82.9)	0.61 (0.31, 1.22)
Employed part-time	98 (71.5)	0.73 (0.47, 1.13)	114 (83.2)	1.28 (0.77, 2.13)	105 (76.6)	1.26 (0.80, 1.98)	123 (89.8)	1.11 (0.59, 2.09)
Other ^a^	324 (77.5)	Reference	332 (79.4)	Reference	302 (72.3)	Reference	371 (88.8)	Reference
Maternal parity, n (%)								
Primiparous	244 (76.0)	0.93 (0.64, 1.33)	253 (78.8)	1.21 (0.82, 1.80)	242 (75.4)	0.88 (0.61, 1.26)	282 (87.9)	1.09 (0.67, 1.78)
Multiparous	226 (74.6)	Reference	248 (81.9)	Reference	221 (72.9)	Reference	269 (88.8)	Reference
Number of children living in household, n (%)								
One	211 (74.6)	Reference	236 (83.4)	Reference	- ^c^	- ^c^	- ^c^	- ^c^
Two	162 (81.0)	1.45 (0.93, 2.27)	157 (78.5)	0.73 (0.46, 1.15)	- ^c^	- ^c^	- ^c^	- ^c^
Three	71 (74.7)	1.01 (0.59, 1.72)	72 (75.8)	0.62 (0.35, 1.10)	- ^c^	- ^c^	- ^c^	- ^c^
Four or more	26 (56.5)	**0.44 (0.23, 0.84)**	36 (78.3)	0.72 (0.33, 1.54)	- ^c^	- ^c^	- ^c^	- ^c^
Childcare (ECE) outside the home, n (%)								
No	394 (76.3)	Reference	409 (79.3)	Reference	370 (71.7)	Reference	- ^c^	- ^c^
Yes	77 (70.6)	**0.54 (0.35, 0.81)**	93 (85.3)	1.52 (0.86, 2.63)	93 (85.3)	**2.29 (1.30, 4.03)**	- ^c^	- ^c^
Socioeconomic deprivation, n (%) ^b^								
1–3 (low)	147 (81.7)	Reference	139 (77.2)	Reference	126 (70.0)	Reference	155 (86.1)	Reference
4–7	214 (75.9)	0.71 (0.44, 1.13)	231 (81.9)	1.34 (0.84, 2.12)	209 (74.1)	1.23 (0.81, 1.86)	251 (89.0)	1.31 (0.74
8–10 (high)	110 (67.5)	**0.47 (0.28, 0.77)**	132 (81.0)	1.26 (0.74, 2.12)	128 (78.5)	1.57 (0.96, 2.56)	146 (89.6)	1.39 (0.72, 2.67)

Did not meet recommendation: introduction of solids between 5 and <7 months (n = 154), puréed food texture introduced first (n = 123), spoon-fed by adult when solids first introduced (n = 162), iron-rich foods introduced first (n = 73). Odds ratio (95% CI) values with *p* values < 0.05 are bolded. ^a^ includes paid parental leave, unpaid parental leave, and unemployed, ^b^ defined according to the NZ Index of Deprivation using participant home address [36], ^c^ less than 10% of the sample was in one of the adherence groups for “number of children in the household” and “childcare (ECE) outside of the home” for the spoon-feeding and iron-rich foods recommendations, which was deemed too small for reliable logistic regression analyses.

**Table 7:** Sociodemographic characteristics associated with adherence to food variety recommendations on both 24-h recall days.

	Iron-Rich Food Consumed on Both Recall Days		Vegetables Consumed on Both Recall Days		Fruit Consumed on Both Recall Days	
	Met Recommendation n = 179	Odds Ratio (95% CI)	Met Recommendation n = 388	Odds Ratio (95% CI)	Met Recommendation n = 388	Odds Ratio (95% CI)
Parent/caregiver age, mean (SD) years						
	33.6 (4.6)	**1.06 (1.02, 1.10)**	33.3 (4.3)	**1.07 (1.04, 1.12)**	33.0 (4.3)	1.03 (1.00, 1.06)
Highest parent/caregiver qualification						
School	20 (22.7)	0.60 (0.35, 1.03)	43 (48.9)	**0.42 (0.27, 0.68)**	35 (39.8)	**0.47 (0.29, 0.75)**
Polytechnic or similar	27 (22.1)	**0.58 (0.36, 0.94)**	66 (54.1)	**0.52 (0.35, 0.79)**	60 (49.2)	0.68 (0.46, 1.03)
University	132 (32.8)	Reference	279 (69.2)	Reference	236 (58.6)	Reference
Employment status of parent/caregiver						
Employed full-time	20 (29.4)	1.07 (0.61, 1.88)	46 (67.6)	1.31 (0.76, 2.26)	33 (48.5)	0.78 (0.47, 1.30)
Employed part-time	44 (32.4)	1.22 (0.81, 1.87)	90 (66.2)	1.22 (0.82, 1.84)	74 (54.4)	0.99 (0.47, 1.31)
Other ^a^	115 (28.1)	Reference	252 (61.5)	Reference	224 (54.6)	Reference
Maternal parity, n (%)						
Primiparous	80 (25.4)	**1.44 (1.01, 2.04)**	178 (56.5)	**1.81 (1.29, 2.52)**	165 (52.4)	1.14 (0.83, 1.57)
Multiparous	98 (32.9)	Reference	209 (70.1)	Reference	166 (55.7)	Reference
Number of children living in household, n (%)						
One	93 (33.6)	Reference	194 (70.0)	Reference	151 (54.5)	Reference
Two	51 (25.6)	0.68 (0.46, 1.02)	126 (63.3)	0.74 (0.50, 1.09)	112 (56.2)	1.07 (0.74, 1.55)
Three	23 (24.5)	0.64 (0.38, 1.10)	52 (55.3)	**0.53 (0.33, 0.86)**	49 (52.1)	0.91 (0.57, 1.45)
Four or more	12 (27.9)	0.77 (0.37, 1.56)	15 (34.9)	**0.23 (0.12, 0.45)**	18 (41.9)	0.60 (0.31, 1.15)
Childcare (ECE) outside the home, n (%)						
No	144 (28.4)	Reference	319 (62.8)	Reference	277 (54.5)	Reference
Yes	35 (33.0)	1.24 (0.80, 1.95)	69 (65.1)	1.10 (0.71, 1.71)	54 (50.9)	0.87 (0.57, 1.32)
Socioeconomic deprivation, n (%) ^b^						
1–3 (low)	56 (31.6)	Reference	125 (70.6)	Reference	103 (58.2)	Reference
4–7	82 (29.6)	0.91 (0.60, 1.37)	180 (65.0)	0.77 (0.51, 1.16)	156 (56.3)	0.93 (0.63, 1.36)
8–10 (high)	41 (25.6)	0.74 (0.46, 1.20)	83 (51.9)	**0.45 (0.29, 0.70)**	72 (45.0)	0.59 (0.38, 0.91)

Did not meet recommendation: iron-rich food consumed on both recall days (n = 179), vegetables consumed on both recall days (n = 388), fruit consumed on both recall days (n = 388). Odds ratio (95% CI) values with *p* values <0.05 are bolded. ^a^ includes paid parental leave, unpaid parental leave, and unemployed, ^b^ defined according to the NZ Index of Deprivation using participant home address [36].

**Table 8:** Sociodemographic characteristics associated with adherence to the recommendation of not adding salt and sugar.

	No Added Salt		No Added Sugar	
	Met Recommendation n = 455	Odds Ratio (95% CI)	Met Recommendation n = 546	Odds Ratio (95% CI)
Parent/caregiver age, mean (SD) years				
	32.7 (5.0)	0.99 (0.95, 1.03)	32.8 (4.9)	**0.94 (0.88, 0.99)**
Highest parent/caregiver qualification				
School	63 (69.2)	1.47 (0.88, 2.44)	74 (81.3)	**3.20 (1.65, 6.19)**
Polytechnic or similar	93 (76.9)	1.00 (0.61, 1.62)	109 (90.1)	1.53 (0.75, 3.14)
University	298 (76.8)	Reference	362 (93.3)	Reference
Employment status of parent/caregiver				
Employed full-time	53 (79.0)	0.85 (0.45, 1.60)	- ^c^	- ^c^
Employed part-time	92 (71.9)	1.26 (0.81, 1.98)	- ^c^	- ^c^
Other ^a^	310 (76)	Reference	- ^c^	- ^c^
Maternal parity, n (%)				
Primiparous	213 (70.1)	**0.53 (0.36, 0.78)**	274 (90.1)	0.84 (0.48, 1.47)
Multiparous	241 (81.4)	Reference	271 (91.6)	Reference
Number of children living in household, n (%)				
One	223 (80.8)	Reference	- ^c^	- ^c^
Two	136 (72.0)	**1.64 (1.06, 2.54)**	- ^c^	- ^c^
Three	63 (69.2)	**1.87 (1.09, 3.18)**	- ^c^	- ^c^
Four or more	32 (72.7)	1.58 (0.76, 3.28)	- ^c^	- ^c^
Childcare (ECE) outside the home, n (%)				
No	376 (75.5)	Reference	453 (91.0)	Reference
Yes	79 (76.7)	0.94 (0.57, 1.54)	93 (90.3)	1.08 (0.53, 2.23)
Socioeconomic deprivation, n (%) ^b^				
1–3 (low)	133 (76.9)	Reference	158 (91.3)	Reference
4–7	203 (75.2)	1.10 (0.70, 1.72)	257 (95.2)	0.53 (0.23, 1.15)
8–10 (high)	119 (75.3)	1.09 (0.66, 1.81)	131 (82.9)	2.17 (1.11, 4.25)

Did not meet recommendation: no added salt (n = 146), no added sugar (n = 55). Odds ratio (95% CI) values with *p* values <0.05 are bolded. ^a^ includes paid parental leave, unpaid parental leave, and unemployed, ^b^ defined according to the NZ Index of Deprivation using participant home address [36], ^c^ less than 10% of the sample was in one of the adherence groups for “employment” and “number of children in the household” for the no added sugar recommendation, which was deemed too small for reliable logistic regression analyses.

**Table 9:** Sociodemographic characteristics associated with adherence to self-feeding from a young age recommendation.

	Not Fully Spoon-Fed at Current Age	
	Met Recommendation n = 543	Odds Ratio (95% CI)
Parent/caregiver age, mean (SD) years		
	32.6 (4.8)	0.99 (0.94, 1.03)
Highest parent/caregiver qualification		
School	79 (84.0)	0.64 (0.34, 1.21)
Polytechnic or similar	103 (82.4)	0.57 (0.33, 1.00)
University	361 (89.1)	Reference
Employment status of parent/caregiver		
Employed full-time	60 (85.7)	1.04 (0.51, 2.15)
Employed part-time	127 (92.7)	**2.21 (1.10, 4.44)**
Other ^a^	356 (85.2)	Reference
Maternal parity, n (%)		
Primiparous	282 (87.9)	0.84 (0.53, 1.33)
Multiparous	260 (85.8)	Reference
Number of children living in household, n (%)		
One	247 (87.3)	Reference
Two	179 (89.5)	1.24 (0.70, 2.20)
Three	82 (86.3)	0.92 (0.47, 1.82)
Four or more	34 (73.9)	**0.41 (0.20, 0.87)**
Socioeconomic deprivation, n (%) ^b^		
1–3 (low)	162 (90.0)	Reference
4–7	242 (85.8)	0.67 (0.37, 1.21)
8–10 (high)	139 (85.3)	0.64 (0.34, 1.23)

Did not meet recommendation (n = 82). Odds ratio (95% CI) values with *p* values < 0.05 are bolded. ^a^ includes paid parental leave, unpaid parental leave, and unemployed, ^b^ defined according to the NZ Index of Deprivation using participant home address [36].

## Data Availability

The data used in the present study are not publicly available due to ethical restrictions related to the consent provided by participants. An ethically compliant dataset may be made available by the corresponding author upon reasonable request and upon approval by the Health and Disability Ethics Committees New Zealand.
